# Coordinating Centers as a Strategy for Accelerating Cancer Epidemiology Consortia: Best Practices

**DOI:** 10.1007/s40471-022-00282-z

**Published:** 2022-02-21

**Authors:** Amy Trentham-Dietz, Jennifer E. Bird, Ronald E. Gangnon, Sara M. Lindberg, Tena Madison, Kristen M. C. Malecki, James D. Shull, Claudia Vredeveld, Betsy Rolland

**Affiliations:** 1 Department of Population Health Sciences, School of Medicine and Public Health, University of Wisconsin-Madison, WARF Room 307, 610 Walnut St., Madison, WI USA; 2 School of Medicine and Public Health, University of Wisconsin Carbone Cancer Center, University of Wisconsin-Madison, Madison, WI USA; 3 Department of Biostatistics and Medical Informatics, School of Medicine and Public Health, University of Wisconsin-Madison, Madison, WI USA; 4 Office of Strategic Consulting, University of Wisconsin-Madison, Madison, WI USA; 5 McArdle Laboratory for Cancer Research, Department of Oncology, School of Medicine and Public Health, University of Wisconsin-Madison, Madison, WI USA; 6 School of Medicine and Public Health, Institute for Clinical and Translational Research, University of Wisconsin-Madison, Madison, WI USA

**Keywords:** Coordinating centers, Team science, Cancer epidemiology

## Abstract

**Purposeof Review:**

This review highlights six “best practices” for cancer epidemiology coordinating centers to facilitate the success of a research consortium.

**Recent Findings:**

Evidence from emerging literature regarding the Science of Team Science suggests that coordinating centers can more effectively foster collaborative cancer epidemiology research in consortia by (1) establishing collaboration as a shared goal at the start, (2) providing scientific expertise complementary to the research sites that adapts over the course of the project, (3) enacting anti-racist and inclusive approaches in all consortium decisions and activities, (4) fostering early-stage investigator career development, (5) engaging stakeholders including cancer survivors as peers, and (6) delivering reliable logistical support and technology tools with planned process evaluation so that researchers can collaboratively focus on the science.

**Summary:**

By drawing on the Science of Team Science, coordinating centers can accelerate research progress and increase the impact of cancer epidemiology consortia.

## Introduction

Team science, particularly for leading chronic conditions like cancer, is increasingly necessary to advance research and research translation. Often cancer epidemiology research consists of teams of collaborators working within consortia for a common scientific purpose. Collaborative teams can often better address large, complex challenges in science, with greater resultant impact, than a single laboratory led by a single principal investigator. Contemporary scientists typically specialize within fields such as cancer epidemiology and/or subfields such as breast cancer epidemiology. These complex areas of research necessitate large research teams to ensure all relevant disciplines are represented, for example, epidemiology as well as primary care and oncology, biostatistics, economics, systems engineering, genetics, and molecular biology, among others. This shift is reflected in the common use of the multiple principal investigator option on research grants and papers with multiple “first” and “last” authors [1]. These teams, while crucial to advancing science, come with logistical challenges.

Within the National Institutes of Health, including the National Cancer Institute (NCI), coordinating centers are used as a strategy to increase the effectiveness of collaborative research consortia. This approach, initially used for large clinical trials, is increasingly applied to observational cancer epidemiology consortia as the numbers of teams and research sites have increased. The number of coordinating centers funded by NCI for research consortia not focused on therapeutic clinical trials has grown over the past decade, with budgets for these coordinating centers increasing over the past 5 years (median $781,467, range $39,592–$14,488,060 across 115 grants during March 2015–March 2021) compared with the previous 5 years (median $608,875, range $5,617–$2,186,017 across 86 grants during March 2009–February 2015) [2]. Transdisciplinary consortia can uniquely develop insights into complex cancer-related scientific questions, but they require dedicated effort to establish common ground, build trust, overcome potentially ingrained prejudices against other fields of study or methods, and inspire collaboration. Despite potential benefits, few examples of best practices to guide coordinating centers exist.

Coordinating centers have the potential to impact many aspects of collaborative research. They can accelerate progress by consortium investigators if the coordinating center handles logistical and administrative tasks allowing the scientists to spend more time on research tasks [3]. Large, complex collaborative teams require greater attention to coordination and communication to minimize redundancy or delays. For example, collaborative teams ultimately produce more publications than projects led by a single investigator, but collaborative publications take slightly longer to accumulate [1, 4]. Efforts must be made to overcome challenges posed by teams that are geographically dispersed around the country, if not the globe [1]. The Science of Team Science is a young discipline that aims to identify strategies for increasing the effectiveness of “research conducted by more than one individual in an interdependent fashion” [1]. Thus, if consortia and coordinating centers can implement the evidence-based lessons learned from the Science of Team Science, they will be more likely to achieve their goals.

The responsibilities of a coordinating center depend on the specific characteristics of the consortium and its scientific scope [3, 5]. Typical activities can include (1) fostering collaboration, (2) facilitating communication and information sharing, (3) completing administrative and logistical tasks, such as planning webinars and in-person meetings, (4) conducting data collection and harmonization, (5) completing coordinated statistical analysis, (6) serving as a central biorepository, (7) managing pilot grant programs, and (8) providing specialized scientific expertise. While many tasks are common to all (or almost all) coordinating centers, such as serving as a repository for policies and planning meetings, other tasks will be tailored to the specific scientific goals and needs of the team members.

**Fig. 1:**
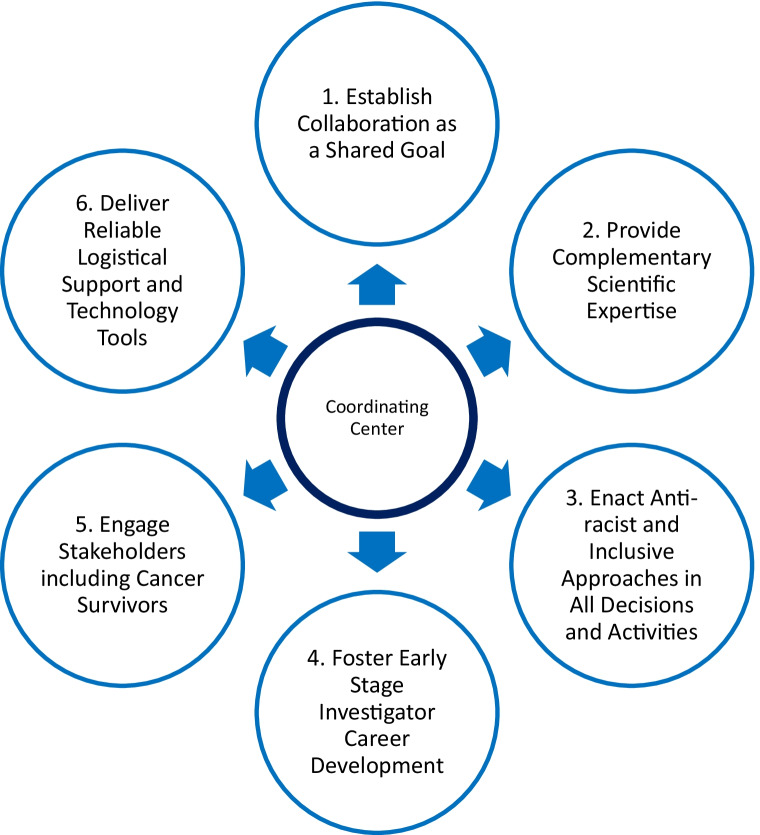
Best practices of an effective coordinating center

This review highlights six “best practices” for cancer epidemiology coordinating centers to facilitate the success of the consortium (Fig. 1). Although this report is based on practical experience with studies involving cancer epidemiology, the best practices draw from the team science literature [1, 6••] and are relevant to coordinating centers and research teams outside of cancer epidemiology.

### Establish Collaboration as a Shared Goal

NCI has recently funded two types of research consortia: ones in which a group of investigators a priori agree to a collaborative approach or ones that are more independent from the outset but required once funded to collaborative. For the a priori approach, principal investigators, often located at multiple institutions, submit a single grant application with a cohesive set of specific aims or projects (e.g., program project awards [7], the Cancer Intervention and Simulation Modeling Network [CISNET] [8], Population-based Research to Optimize the Screening Process [PROSPR] second funding cycle [9]). For the latter independent approach, each research site submits autonomous grant applications frequently with disparate specific aims (e.g., Breast Cancer and the Environment Research Program [BCERP] [10], PROSPR first funding cycle, Cohorts for Environmental Exposures and Cancer Risk [CEECR] [11]). The independent approach can result in limited overlap in scientific scope and methods with respect to recruitment plans, sampling frames, biomarker choices, exposure assessments, and health outcomes across the funded projects. This lack of overlap in initial study design and aims is a significant barrier to cross-consortium collaborative research. Investigators are sometimes reluctant to share preliminary research, and the availability of pilot grant awards and timing of such can be insufficient to motivate collaboration. True large-scale collaboration requires commitment to a shared mission from the outset. Administrative policies at the institutional and funding agency levels can restrict flexibility for re-budgeting projects to increase commonalities across research sites, and investigators may be resistant to adopting common methods and scientific goals in favor of their own independent pursuits. Thus, coordinating centers can serve an essential function by working with the funding agency’s representatives to clarify expectations for collaboration (and consequences for nonparticipation) with researchers and guide them through consensus development.

While an informal goal-setting process may be adequate to establish consensus regarding expectations for collaboration for some teams, consortia may benefit from developing a formal Collaboration Plan. The potential benefit of a short (90-min) intervention designed to assist translational teams as they conduct collaboration planning is currently being evaluated [12]. As described by Hall et al. [13], a formal Collaboration Plan is a “living document” that provides a framework for discussions and planning for collaboration surrounding ten “key influences”: (1) rationale for team approach and configuration; (2) collaboration readiness; (3) technological readiness; (4) team functioning; (5) communication and coordination; (6) leadership, management, and administration; (7) conflict prevention and management; (8) training; (9) quality improvement activities; and (10) budget and resource allocation. Consortium members benefit when, during this time of goal setting and collaboration planning, coordinating centers are transparent and provide clarity around the services and support available from the coordinating center itself.

### Provide Complementary Scientific Expertise

A coordinating center serving as the intellectual hub for a consortium can provide expertise complementary to consortium members, e.g., technical training for laboratory methods, study design and methods, stakeholder engagement, questionnaire development, study protocol harmonization, and dissemination of results. Many scientists do not have training, time, or interest in ensuring research findings reach lay audiences or advocates. Coordinating centers have a unique opportunity to facilitate more effective and faster dissemination and translation by providing implementation science expertise. Individual research sites may not fully take advantage of expertise available at a coordinating center instead drawing on their own teams and often duplicating efforts. Ideally, expertise at a coordinating center should enhance and integrate the science at individual sites. Furthermore, a consortium will benefit if a coordinating center has the flexibility to add (and drop) expertise over time.

**Fig. 2:**
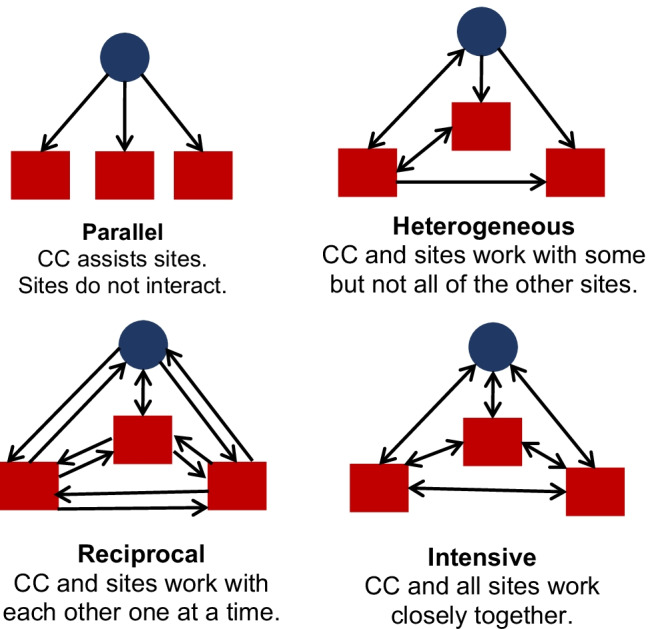
Schema of various levels for the intensity of consortium interactions between a coordinating center and research sites. Coordinating centers represented by blue circles and research sites represented by red squares. Abbreviation: CC, coordinating center. Adapted from Kozlowski and Bell [15]

Team science has a greater need for overall organization, integration, and inter-dependency than individual investigator-led projects. The greater complexity demands contributions from multiple team members. In fact, a large group of individuals is not necessarily a “team”; instead, a team requires that individuals interact meaningfully towards a common goal [14]. One person working alone cannot accomplish the team’s goals, and, conversely, budget and other practical restrictions require that all team members participate. Some consortia may only require that groups of investigators work in parallel, whereas other consortia necessitate greater coordination and integration [1, 15]. Thus, coordinating centers should adjust the support and tools they provide to the consortium’s needs and complexity. For example, a consortium with investigators that work mostly in parallel may only need monthly meetings for efficient coordination, whereas other consortia with reciprocal dependencies across investigators, heterogeneously interactive, or intensively interdependent researchers require frequent asynchronous communication between meetings, e.g., email and direct messaging, secure websites to store shared files, and careful documentation of decisions and workflows (Fig. 2) [15].

Ideally, coordinating centers will adapt their expertise and technology tools to address the most significant needs of the research sites; consequently, research sites may also benefit by relying on coordinating centers for the services they offer, e.g., statistical analysis, even when the sites originally intended to conduct those activities locally [1]. One challenge scientists applying for consortium-based grants face is they may not know what type of support a coordinating center will provide and thus what areas require funds in their own application budgets. For example, BCERP sites were unaware of the biostatistics services available at the coordinating center when they submitted their applications so they needed to budget for all necessary statistical expertise in their own individual applications. Future consortia may benefit if a coordinating center was funded in advance of potential research sites to lead hypothesis building and harmonization activities before the research is initiated by the investigators and so that the researchers will know what support services will be provided by the coordinating center from the outset. On the other hand, if research sites were funded in advance of the coordinating center, the coordinating center would be able to more closely align their services to the needs of the consortium members.

Coordinating centers are uniquely able to provide integration of team science experts such as a Consortium Director or Team Science Facilitator into the team. These individuals can focus “essentially exclusively on the time-consuming and challenging team-science aspects of the collaborative project, combining scientific and administrative knowledge and experience with excellent people skills and a deep understanding of how collaboration is actually managed” [16]. These professionals, generally doctorally trained researchers with background knowledge or expertise in the Science of Team Science, lead efforts in strategic planning, facilitating meetings, and leading program evaluation, among other key team science activities. This team member could also advise sites on their own collaborative and team processes.

Cancer research consortia fall across the entire spectrum of cross-disciplinarity. Multidisciplinary (more than one discipline mostly working separately), interdisciplinary (integration and synergy across approaches), and transdisciplinary (unified collaboration beyond individual perspectives) interaction between epidemiologists, laboratory scientists, clinicians, and community stakeholders is a hallmark of many cancer consortia [17, 18]. Groups of researchers and their community partners often approach scientific challenges from varied perspectives, which can lead to conflict in the absence of mutual respect, and friction when goals and collaborative partnerships are not well-established or grounded in trust. Success at building trust and avoiding conflict requires consortium members to be open, and to demonstrate their openness, to new approaches and to agree upon a mutual understanding of terminology [1]. Coordinating centers are uniquely positioned to guide consortia with high diversity in disciplines through this “conceptualization” phase of team science [4]. Two approaches for facilitating trust include planning informal opportunities for consortium members to connect on a personal level, for example, a group meal or social event, and formal opportunities for members to understand each other’s working or learning styles. Coordinating centers can also provide consortium members with training in self-correction (identify areas for improvement) and to better prepare as a team for disruptions [1]. By adapting their support of the consortium throughout the lifespan of a research project, a coordinating center can assist consortium members to overcome threats to trusting collaborations between members of different disciplines [19].

### Enact Anti-racist and Inclusive Approaches in all Decisions and Activities

Just as diversity in disciplines generates scientific benefits in consortia, so too does demographic diversity in research teams [20]. However, there are long-standing disparities in success rates experienced by women and Black applications for R01-equivalent research project grants [21, 22]. While NIH defines diversity for their extramural programs in terms of groups underrepresented in science—including individuals from underrepresented racial and ethnic groups, individuals with disabilities, and individuals from disadvantaged backgrounds—diversity for health disparities also includes groups based on culture, religion, sex and gender, and geographic residence [23]. Diversity is essential to all components of the consortia including the coordinating center, research sites, and grant agency project scientists as well as the grant review panels. Decisions about scientific priorities and use of resources must not be made by groups lacking diversity. Thus, coordinating centers must play a central role in creating collaborations that are more equitable by fostering access to resources and leadership positions in consortia and by facilitating the inclusion of diverse members in all roles and at all career stages [24].

Beyond representation among researchers, representation of communities carrying disproportionate burdens of cancer is also required to bring about the innovation and creative breakthroughs necessary to eliminate disparities. Coordinating centers are responsible for creating an environment that creates common ground, fosters relationships, retains diverse members, and welcomes creative tension. The creation of such an environment should be explicit and part of the planning process. Project-related materials, for example, the consortium website, need to reflect this capacity for coordinating centers to serve and foster diverse working groups and engage with community partners from various backgrounds, non-government organizations, and policy makers. The work involved in preparing the way for diversity begins at a personal and collective level but also structurally with communication of core values or guiding principles, policies, and forums for inclusive or consultative decision making. Friction resulting from the inclusion of differences of opinions and perspectives provides the fuel to catalyze innovation that would not occur among more homogeneous teams [1, 20]. Coordinating center leaders and meeting facilitators need to foster environments intolerant of racism and manage the level of creative tension, so all members feel secure in participating in respectful dialogue and expressing disagreement without generating serious conflict that may lead to erosion in trust [1].

### Foster Early-Stage Investigator Career Development

Incentives in academic research often conflict with principles of team science including publication conventions and promotion guidelines. This is particularly true for mentoring and career development. Lead scientists typically face tension between their desire to support career development and mentoring for early stage investigators (ESIs) and their primary responsibilities for the success (and continued funding) of their scientific projects. Conventions are evolving with some universities expanding tenure guidelines to include contributions to team science as evidence of scholarship and impact [1, 25, 26]. Coordinating centers can foster the career development of ESIs by providing training in evidence-based approaches for navigating the beginning phases of a career in science. Formal mentorship education that cultivates inclusive practices in mentoring is more effective than informal mentoring approaches [27]. *Mentoring Up*, an example of an evidence-based mentoring curriculum, helps ESIs develop knowledge and skills to proactively and effectively navigate their mentoring relationships and career progression [28]. Interacting with best practice #3 described above (“enact anti-racist practices”), coordinating centers can also promote culturally aware mentoring training for both mentors and mentees [29].

Other strategies for supporting ESIs include (1) prioritizing ESI for first-author positions on consortium publications, (2) reserving ESI voting member positions in consortium committees including the Steering Committee, (3) planning opportunities for peer-networking at consortium meetings, and (4) distributing travel awards supporting ESI attendance at consortium meetings and scientific conferences. Consortia provide many opportunities for ESIs to expand their network of peers and mentors beyond their local institutions. Supported by a coordinating center, consortia can provide both a critical mass of peers and training in cross-disciplinary collaboration skills and knowledge exchange, core competencies that are essential to population health science [30]. Due to their major role in planning consortium meetings, coordinating centers can uniquely increase the visibility of ESIs and provide a supportive environment for developing oral presentation skills. Supporting the development of ESIs leads to greater sustained scientific impact of the consortium that continues long after a limited grant project period.

### Engage Stakeholders Including Cancer Survivors

Engaging stakeholders from affected communities within cancer research consortia is crucial to ensuring effective research translation. Cancer research stakeholders include cancer survivors, caregivers and family members, members of cancer advocacy organizations, and medical professionals that treat cancer patients. Stakeholder input increases opportunities to expand the relevance of the science to communities at risk and has many practical benefits such as supporting achievement of recruitment goals and improving program sustainability [31]. Coordinating centers need to foster bi-directional communication by overcoming communication barriers. This can be as simple as providing individual assistance to stakeholders including technology support for participating in conference calls and web meetings, transportation, and education regarding academic research. Travel funds distributed by coordinating centers may be instrumental for including stakeholders in consortium meetings. Training workshops for consortia members can support integrating stakeholders as full members of research teams, for example, training for researchers on designing for dissemination [32]. Workshops can also assist researchers with deep engagement of stakeholders across all phases of a research project, from pre-planning through dissemination [33]. Most importantly, these workshops facilitate the development of strong and mutually beneficial relationships between scientists and community partners, keeping community partners invested in the research project and minimizing turnover of community partners.

### Deliver Reliable Logistical Support and Technology Tools

Communication, coordination, and project management are keys to successful collaborative work. Communication between multiple investigators at institutions across the USA, if not the world, requires consortium members to participate in regularly scheduled remote meetings. Use of online video meetings, which dramatically increased during 2020 due to the COVID-19 pandemic, will likely continue as the preferred choice for remote meetings. In addition to synchronous communication, consortium members also need access to a shared knowledge base, commonly through a secure website [34]. Substantial amounts of staff time are typically required to create and maintain a secure website, which are often underutilized by consortium members. Conversely, email is used frequently but is a poor medium for interactive, threaded discussions. New integrated technology platforms that offer multiple tools and methods of communication are increasingly available that are user-friendly and secure against inappropriate access [1]. However, consortium members often have varied preferences on use of technology tools, and no single tool will be preferred by everyone. Coordinating centers need to consider consortium member preferences and their specific needs when establishing the technology tools and routes of communication for a consortium, while also realizing the practical limitations of trying to support an unlimited number of pathways. Technology platforms can provide effective methods for consortium members to participate in decision-making and other research activities; coordinating centers can leverage technology tools to more fully engage broader participation by consortium members. Tools provided centrally by a coordinating center can uniquely help researchers to focus on making substantive progress on their scientific objectives rather than the logistical and administrative responsibilities inherent in a research program. Furthermore, they add transparency to the activities of the consortium, aiding in the development of a culture of trust, openness, and inclusivity [19]. By selecting a comprehensive plan for communication and providing user support for the chosen tools, a coordinating center can avoid providing structural support for an unwieldy number of tools used by subsets of the consortium.

Logistical and administrative tasks including meeting planning and routine process evaluations are critical for the science to happen effectively. All major research organizations employ staff that can efficiently and reliably execute administrative tasks such as scheduling and planning meetings, taking and circulating meeting notes, tracking milestones, and establishing subcontracts. Competent performance of these routine tasks is essential to the success of the consortium. Chronic small errors or lack of support from the coordinating center can quickly erode the willingness of consortium members to devote time to collaborative activities. Administrative coordinating skills are often over looked as essential components, but resources and time in budgeting for strong administrative support in coordinating centers are critical.

Similarly, meeting planning skills are essential for any coordinating center. Ensuring consortium members are informed of meeting goals in advance ensures that researchers can use their time more efficiently by participating in activities where their input is beneficial and avoiding activities where their input is not needed [34]. Importantly, coordinating centers can implement a variety of formats for meetings—other than the traditional format where a small number of speakers dominate a discussion—that are specifically designed to achieve meeting goals. For example, a meeting designed to identify novel priority ideas for collaborative pilot projects (e.g., a “World Café” or “sand pit”/idea laboratory format) should be facilitated differently than a meeting intended for members to become familiar with the research interests of other members (e.g., speed networking) [35, 36]. Finally, coordinating centers should routinely survey consortium members regarding their satisfaction throughout a project period. Online questionnaires administered immediately after annual consortium meetings are an important source of data for informing a wide variety of topics including the formats of future meetings and identification of stakeholders or areas of expertise that may benefit members if added to the consortium. Formal evaluation efforts by a coordinating center can identify additional opportunities to improve their effectiveness at helping consortium members to achieve their scientific goals.

## Conclusions

An effective coordinating center can be viewed as the implementation of a bundle of interventions to improve research outcomes of team science. Efforts to improve the full breadth of coordinating center activities can be informed by evidence-based best practices and not just historical precedent [37]. Coordinating centers may be responsible for a wide range of activities from arranging logistics for in-person collaborative meetings to participating as a scientific peer in the research. However, emerging scholarship demonstrates that the Science of Team Science, when applied to cancer epidemiology consortia through coordinating centers, hold potential for substantively accelerating research progress and increasing impact of the research products. As such, we highly encourage all consortia to engage the support of experts in the Science of Team Science as key members of the coordinating center, thus ensuring the application of evidence-based approaches to team science facilitation.
